# Glucose–lipid metabolic dysregulation and sleep fragmentation in obstructive sleep apnea: insights from a large-scale cross-sectional study and exploratory hypoxia-related single-nucleus transcriptomic analysis

**DOI:** 10.3389/fnut.2026.1859107

**Published:** 2026-07-10

**Authors:** Zhicheng Wei, Cheng Lu, Jingchun He, De Huai, Jinhong Shen, Jian Guan, Shankai Yin

**Affiliations:** 1 Department of Otorhinolaryngology Head and Neck Surgery, Shanghai Sixth People’s Hospital Affiliated to Shanghai Jiao Tong University School of Medicine, Shanghai, China; 2 Shanghai Key Laboratory of Sleep Disordered Breathing, Shanghai, China; 3 Otorhinolaryngology Institute of Shanghai Jiao Tong University, Shanghai, China; 4 Department of Otorhinolaryngology-Head& Neck Surgery, Xinhua Hospital, Shanghai Jiaotong University School of Medicine, Shanghai, China; 5 Shanghai Jiaotong University School of Medicine Ear Institute, Shanghai, China; 6 Shanghai Key Laboratory of Translational Medicine on Ear and Nose Diseases, Shanghai, China; 7 Department of Otorhinolaryngology Head and Neck, The Affiliated Huai’an Hospital of Xuzhou Medical University, Huaian, China

**Keywords:** chronic intermittent hypoxia, glucose–lipid metabolism, obstructive sleep apnea, single-nucleus transcriptomics, sleep fragmentation, TyG index

## Abstract

**Background:**

Sleep fragmentation impairs health and quality of life, yet its relationship with glucose–lipid metabolic dysregulation remains unclear. This study investigated this association in obstructive sleep apnea (OSA), with a focus on nocturnal hypoxia and hypoxia-related brainstem cellular responses.

**Methods:**

This large-scale cross-sectional study included 5,885 adults with suspected OSA from the Shanghai Sleep Health Study. Glucose–lipid metabolic status was assessed using the triglyceride–glucose (TyG) index, TyG-body mass index (TyG-BMI), metabolic score for insulin resistance (METS-IR), and metabolic syndrome. Sleep fragmentation was quantified by the micro-arousal index (MAI). Multivariable regression, restricted cubic spline, threshold effect, multiplicative and additive interaction, stratified analyses, Benjamini–Hochberg false discovery rate (FDR) correction were performed. GEO single-nucleus analyses were conducted to explore intermittent hypoxia-related cellular and metabolic pathway responses in the brainstem (metabolic activity scoring, GSVA, scTenifoldKnk).

**Results:**

TyG, TyG-BMI, and METS-IR were independently positively associated with MAI. Each 1-unit increase in TyG was associated with a 4.0-unit increase in MAI (*β* = 4.0, *p* < 0.001). TyG-BMI and METS-IR showed significant nonlinear dose–response relationships with thresholds at 310.31 and 57.25, respectively. OSA severity and nocturnal hypoxia significantly strengthened these associations through both multiplicative and additive interactions. When AHI > 5, the associations of TyG (*β* = 4.04), TyG-BMI (β = 0.08), and METS-IR (β = 0.41) on MAI were more pronounced. Combined exposure was associated with 4.07–5.91-fold higher odds of elevated MAI. All primary associations remained significant after FDR correction. Single-nucleus analyses suggested widespread glucose–lipid metabolic reprogramming in brainstem cells, particularly neurons and oligodendrocytes.

**Conclusion:**

Glucose–lipid metabolic abnormalities were significantly associated with OSA-related sleep fragmentation, and these associations were stronger among participants with greater OSA severity and nocturnal hypoxia.

## 1 Introduction

Obstructive sleep apnea (OSA) is a highly prevalent yet underdiagnosed sleep-disordered breathing (SDB). It is estimated to affect approximately 936 million adults worldwide, of whom over 400 million suffer from moderate to severe disease, making it a major global public health concern (1, 2). In recent years, it has been increasingly recognized as a systemic disease closely associated with metabolic dysregulation (3, 4). A large body of evidence indicates that OSA patients frequently present with obesity, dysregulation of glucose and lipid metabolism, insulin resistance, and metabolic syndrome. Moreover, these metabolic abnormalities progressively worsen with increasing OSA severity (5, 6).

The core pathophysiological features of OSA include not only chronic intermittent hypoxia (CIH) but also disruption of sleep continuity and architecture, manifested as sleep fragmentation. Sleep fragmentation, as quantified by the micro-arousal index (MAI) and can lead to excessive daytime sleepiness, cognitive decline, and neurobehavioral disturbances, thereby markedly impairing patients’ quality of life (7, 8). However, sleep fragmentation has traditionally been viewed as a downstream consequence of respiratory events in OSA, and whether glucose–lipid metabolic abnormalities are associated with sleep fragmentation remains poorly understood (9).

In recent years, a range of composite indices of glucose–lipid metabolism has attracted increasing attention, including the triglyceride–glucose index (TyG index), the TyG–BMI index, and the metabolic score for insulin resistance (METS-IR) (10–13). These indicators are considered reliable proxies for insulin resistance, offering higher stability and accessibility than traditional measures, and can more comprehensively reflect the body’s glucolipid metabolic status. Studies have found these indicators to be closely associated with the development and progression of OSA (14–17). However, their relationships with sleep architecture, particularly sleep fragmentation, remain insufficiently understood.

Numerous animal and clinical studies have confirmed that CIH serves as a key driver of glucose–lipid metabolic disturbances such as peripheral insulin resistance and dyslipidemia (18, 19). However, OSA-related glucose–lipid metabolic abnormalities and sleep fragmentation often coexist, and the relationship between them is intricate and complex (3, 20, 21). It remains unclear whether CIH influences sleep fragmentation through intricate interactions with glucose–lipid metabolic dysfunction.

Sleep homeostasis depends on the fine regulation of the central nervous system, particularly the brainstem region (22, 23). Recent studies suggest that CIH can affect the function of both neurons and glial cells and may also influence metabolic processes in the brain (24–27). Based on this, we hypothesize that CIH may be associated with sleep fragmentation through alterations in glucose and lipid metabolism in the brain, potentially accompanied by changes in neuronal and glial cell function.

Accordingly, this study adopts a glucose–lipid metabolism–related perspective on sleep fragmentation and integrates clinical data with exploratory single-nucleus transcriptomic analyses to investigate potential biological associations. Specifically: (1) to examine the associations between glucose–lipid metabolic indices (TyG index, TyG–BMI index, and METS-IR) as well as metabolic syndrome and sleep fragmentation (MAI); (2) to explore whether OSA severity and hypoxia-related indices (AHI, ODI, CT90, and Min SpO_2_) modify this relationship; and (3) to characterize hypoxia-associated transcriptomic and metabolic alterations in brainstem cells using publicly available single-nucleus transcriptomic data from CIH-exposed mice.

## 2 Materials and methods

### 2.1 Patients and study design

From July 2007 to September 2018, a total of 9,172 snoring patients with suspected OSA were consecutively recruited from the Shanghai Sleep Health Study (SSHS). After providing informed consent, participants underwent standardized assessments, including medical history collection, anthropometric measurements, overnight polysomnography (PSG), and blood biochemical testing.

Participants were excluded if they met any of the following criteria: (1) age <18 years; (2) prior treatment for OSA, diabetes, obesity, or dyslipidemia; (3) presence of other sleep disorders (e.g., upper airway resistance syndrome, restless legs syndrome, or narcolepsy); (4) use of antipsychotic drugs, antidepressants, or other medications that could affect sleep; or (5) missing data for key variables or the presence of extreme values. Finally, 5,885 participants were included in the analysis (Supplementary Figure S1).

The study protocol was approved by the Ethics Committee of Shanghai Jiao Tong University Affiliated Sixth People’s Hospital and was conducted in accordance with the Declaration of Helsinki (Clinical trial registration: ChiCTR1900025714). Written informed consent was obtained from all participants.

### 2.2 Anthropometric measurements and metabolic assessments

#### 2.2.1 Anthropometric measurements

Baseline data was collected by trained staff following standardized procedures. Height, weight, waist circumference (WC), neck circumference (NC), and hip circumference (HC) were measured with participants wearing light clothing and no shoes. Body mass index (BMI) was calculated as weight (kg) divided by height squared (m^2^).

After at least 30 min of rest (without exercise, caffeine intake, or smoking), systolic blood pressure (SBP) and diastolic blood pressure (DBP) were measured using a standard mercury sphygmomanometer. Two measurements were taken at least one minute apart, and the average value was recorded. Additional measurements were obtained if the difference between readings exceeded acceptable limits.

Smoking status was categorized as current/former smoker or never smoker. Alcohol consumption was classified similarly.

#### 2.2.2 Metabolic measurements

Fasting blood samples were collected at approximately 7:00 a.m. for biochemical analyses. The lipid profile included total cholesterol (TC), triglycerides (TG), high-density lipoprotein cholesterol (HDL-C), low-density lipoprotein cholesterol (LDL-C), apolipoprotein A1 (ApoA1), apolipoprotein B (ApoB), and apolipoprotein E (ApoE). All measurements were performed in the clinical laboratory of Shanghai Sixth People’s Hospital.

Fasting plasma glucose (FPG) and fasting insulin levels were measured using an automated analyzer (H-7600; Hitachi, Tokyo, Japan). Insulin resistance was estimated using the homeostasis model assessment of insulin resistance (HOMA-IR), calculated as:

HOMA-IR = fasting insulin (μU/mL) × FPG (mmol/L)/22.5 (28).

#### 2.2.3 Construction of glucose–lipid composite indices

In this study, three widely used glucose–lipid-related composite indices were calculated, including TyG, TyG-BMI, and METS-IR, based on FPG, TG, BMI, and HDL-C.

The TyG index was calculated as follows (29):


TyG=ln[TG(mmol/L)×FPG(mmol/L)/2]

The TyG-BMI index was derived by multiplying TyG by BMI (16):


$$\mathrm{TyG}-\mathrm{BMI}=\mathrm{TyG}\times \mathrm{BMI}\;(\mathrm{kg}/m^{2})$$

The METS-IR was calculated using the following formula (17):


METS−IR=[ln(2×FPG+TG)×BMI]/ln(HDL−C)

These indices have been validated as reliable surrogate markers of insulin resistance and metabolic dysfunction.

#### 2.2.4 Definition of metabolic syndrome

Metabolic syndrome was defined as the presence of three or more of the following five components: (1) waist circumference ≥90 cm in men or ≥85 cm in women; (2) triglycerides ≥1.7 mmol/L or treatment; (3) HDL-C < 1.03 mmol/L in men or <1.29 mmol/L in women; (4) blood pressure ≥130/85 mmHg or antihypertensive treatment; (5) fasting plasma glucose ≥5.6 mmol/L or previously diagnosed diabetes. The definition was based on the revised NCEP ATP III criteria with Chinese-specific waist circumference cutoffs (30, 31).

### 2.3 Sleep evaluation

All participants underwent overnight PSG at the Sleep Research Center of Shanghai Sixth People’s Hospital. Environmental conditions, including noise, light, and temperature, were controlled, and participants were acclimated to the sleep laboratory prior to recording.

PSG was performed using standard systems (Alice-5 or Alice-6; Philips Respironics, Pittsburgh, PA, United States) and included electroencephalography, electromyography, electrocardiography, airflow monitoring, respiratory effort assessment, and pulse oximetry. Sleep and respiratory parameters were scored according to the American Academy of Sleep Medicine (AASM) 2007 criteria (32).

Apnea was defined as a ≥ 90% reduction in airflow lasting ≥10 s, and hypopnea was defined as a ≥ 30% reduction in airflow accompanied by a ≥ 3% oxygen desaturation or arousal. The apnea–hypopnea index (AHI) was calculated as the number of apnea and hypopnea events per hour of sleep. OSA was defined as AHI ≥ 5 events/h and categorized as mild (5–15), moderate (15–30), or severe (≥30).

The oxygen desaturation index (ODI) was defined as the number of ≥3% desaturation events per hour of sleep. Minimum peripheral capillary oxygen saturation (minimum SpO₂) and cumulative time spent with SpO₂ < 90% (CT90) were also recorded. Sleep fragmentation was assessed using the MAI, defined as the number of micro-arousals per hour of sleep.

Sleep architecture was evaluated using total sleep time (TST), sleep efficiency (SE; TST/time in bed), and sleep stage distribution, including rapid eye movement sleep (REM/TST, %), stage N1 sleep (N1/TST, %), and stage N3 sleep (N3/TST, %).

### 2.4 Clinical data analysis

The primary outcome of this study was sleep fragmentation, assessed by MAI.

All statistical analyses were performed using R software (version 4.4.3). Normally distributed variables were expressed as mean ± standard deviation, whereas skewed variables were presented as median (interquartile range). Categorical variables were expressed as percentages. Differences among groups were compared using the Kruskal–Wallis test or chi-square test, as appropriate.

Spearman’s rank correlation analysis was performed to assess the correlations between glucose–lipid metabolic indices and sleep architecture parameters, with correlation coefficients and corresponding *p* values calculated and visualized using a correlation heatmap.

Multivariable linear regression models were constructed to assess the associations between glucose–lipid metabolic indices and sleep parameters after adjustment for age, sex, BMI, smoking status, and alcohol consumption. Because BMI is included in the calculation of TyG–BMI and METS-IR, it was not additionally adjusted for in the corresponding models.

Restricted cubic spline (RCS) models with four knots at the 5th, 35th, 65th, and 95th percentiles were fitted using the plotRCS package (version 0.1). The 5th percentile was set as the reference to evaluate potential nonlinear dose–response relationships between glucose–lipid metabolic indices and MAI. Segmented regression models were applied to identify potential threshold effects, and F-tests were used to compare segmented and linear models.

To systematically evaluate the interactions among metabolic indices, OSA, hypoxia-related indices, and MAI, both multiplicative and additive interaction analyses were performed. For multiplicative interactions, interaction terms between metabolic indices and OSA (AHI), as well as hypoxia-related indices (ODI, CT90 and Min SpO_2_), were incorporated into multivariable linear regression models. To facilitate clinical interpretation, AHI (≥5 events/h), ODI (median split), CT90 (median split), and minimum SpO₂ (≤90%) were additionally dichotomized. The low-exposure groups (AHI < 5 events/h, ODI and CT90 below the median, and minimum SpO₂ > 90%) were used as the reference categories. For additive interactions, logistic regression models with MAI dichotomized at the median were used to calculate the relative excess risk due to interaction (RERI), synergy index (S), and attributable proportion, in order to evaluate biological interactions between metabolic indices and OSA/hypoxia.

Stratified analyses by sex and age quartiles were performed to assess the robustness of the associations. In all analyses, a two-sided *p* < 0.05 was considered statistically significant.

To address the issue of multiple comparisons and control the false discovery rate (FDR), Benjamini–Hochberg (BH) correction was applied to all primary hypothesis testing within predefined analysis families. Specifically, multivariable regression analyses, RCS analyses, and interaction analyses were separately grouped and adjusted using the BH procedure. Statistical significance for FDR-controlled analyses was defined as q < 0.05.

### 2.5 Single-nucleus transcriptomic data sources

Single-nucleus transcriptomic data were obtained from the Gene Expression Omnibus (GEO) database (GSE256102), comprising single-nucleus RNA sequencing data from the pons–medulla (brainstem) of male mice exposed to CIH or normoxic conditions (33). Male C57BL/6 J mice were exposed to CIH or normoxia from postnatal day 22 to day 43. The CIH protocol consisted of alternating cycles of hypoxia (4.5–5% O₂) and normoxia (21% O₂), repeated 80 times per day for 8 h daily over 21 days. The dataset included three biological replicates per group.

### 2.6 Single-nucleus transcriptomic data analysis

#### 2.6.1 Clustering and cell annotation

Quality control and downstream analyses were performed using Seurat (Version 5.0). Cells with fewer than 200 detected genes, more than 6,000 detected genes, or >10% mitochondrial transcripts were excluded. Data were normalized using the LogNormalize method with a scale factor of 10,000, and 1,500 highly variable genes were identified using the variance-stabilizing transformation (VST) method.

The data was scaled and subjected to principal component analysis (PCA). Batch effects among samples were corrected using Harmony based on sample identity (orig.ident). The first 20 Harmony-corrected principal components were used for downstream analyses. Cell clustering was performed using the FindNeighbors and FindClusters functions with a clustering resolution of 0.6, and clusters were visualized using t-distributed stochastic neighbor embedding (t-SNE).

Cell type annotation was conducted using the SingleR package (Version 2.8) with the MouseRNAseq and ImmGen reference datasets. Cluster-level annotations generated by SingleR were mapped back to individual cells for downstream analyses and visualization.

#### 2.6.2 Construction of glucose–lipid metabolism gene sets and activity scoring

Gene sets related to glucose and lipid metabolism were obtained from the MSigDB database (m2.all.v2026.1. Mm). A curated gene set was constructed by keyword-based filtering of pathways related to glycolysis, gluconeogenesis, glucose and carbohydrate metabolism, the tricarboxylic acid cycle, pyruvate and lactate metabolism, fatty acid metabolism, cholesterol metabolism, lipid transport, and metabolism-related regulatory signaling pathways. Redundant or biologically irrelevant pathways were excluded, and the remaining gene sets were merged and deduplicated.

At the single-nucleus level, metabolic activity scores were calculated using the AddModuleScore function in Seurat. Group-wise comparisons between Control and CIH groups, as well as cell type–specific analyses, were performed using Wilcoxon rank-sum tests with Benjamini–Hochberg correction.

#### 2.6.3 Gene set variation analysis

Gene set variation analysis (GSVA) was performed to evaluate pathway-level metabolic activity using glucose–lipid metabolism–related gene sets curated with the same keyword-based strategy. Gene sets with fewer than 5 or more than 500 genes were excluded, and only pathways with at least one gene detected in the expression matrix were retained.

For GSVA input, gene expression matrices were generated using the AverageExpression function in Seurat by calculating average expression values across experimental groups. Enrichment scores for each pathway were calculated using the GSVA package with the gsvaParam function and maxDiff = TRUE.

The resulting GSVA enrichment scores were used for visualization of pathway activity. Heatmaps were generated using the pheatmap package (Version 1.0) with row scaling and group annotation.

#### 2.6.4 Cell–cell communication analysis

To investigate intercellular signaling, cell–cell communication analysis was performed using the CellChat package (Version 1.6). The normalized gene expression matrix was extracted from the Seurat object (RNA assay, log-normalized data), and cells were grouped according to annotated cell types.

CellChat objects were constructed separately for Control and CIH groups using cell type as the grouping variable. The CellChat workflow included data subset selection, identification of overexpressed genes and ligand–receptor interactions, projection onto a mouse protein–protein interaction (PPI) network, and computation of communication probabilities.

Intercellular communication networks were summarized based on the number of inferred interactions. Comparative visualization between the Control and CIH groups was performed after merging CellChat objects using the mergeCellChat function. Circle plots were generated to visualize global cell–cell communication networks in each group.

#### 2.6.5 Virtual gene knockout analysis

To explore potential regulatory mechanisms, virtual gene knockout analysis was performed using the scTenifoldKnk package (Version 1.0) (34). The analysis was restricted to CIH samples to capture disease-specific regulatory networks. Raw count matrices were extracted from the Seurat object, and highly variable genes (HVGs) were identified using a variance-stabilizing transformation (VST) approach (top 5,000 genes). The top 2,000 HVGs were combined with the target gene to construct the input gene set.

Gene regulatory networks were inferred using scTenifoldKnk with three subsampled networks (nc_nNet = 3), each constructed using 300 randomly selected cells (nc_nCells = 300). Virtual knockout of the target gene was simulated by perturbing its regulatory influence within the inferred networks.

Differentially regulated genes were identified by comparing network structures before and after perturbation, and statistical significance was assessed using adjusted *p*-values (p.adj < 0.05). Results were ranked according to differential regulation statistics (FC and *Z* scores). Visualization was performed using bar plots and volcano plots to highlight significantly dysregulated genes.

## 3 Results

### 3.1 Basic characteristics

A total of 5,885 snoring patients with suspected OSA were included in this study, predominantly middle-aged males with a mean age of approximately 42 years. Based on the AHI, participants were categorized into Non-OSA, Mild OSA, Moderate OSA, and Severe OSA groups (Table 1). Compared with the Non-OSA group, OSA patients were more likely to be male and exhibited higher BMI, as well as larger NC, WC, and HC. With increasing OSA severity, levels of FPG, fasting insulin, TC, TG, LDL-C, ApoB, and ApoE, as well as the prevalence of metabolic syndrome, progressively increased. Correspondingly, HOMA-IR, TyG index, TyG-BMI index, and METS-IR also showed a stepwise elevation (*p* < 0.001). Overall, these metabolic parameters exhibited a graded, stepwise increase with worsening OSA severity.

**Table 1:** Comparison of basic characteristics among different OSA severity groups.

Characteristic	*N* ^1^	Overall *N* = 5885^2^	Non-OSA *N* = 1067^2^	Mild OSA *N* = 888^2^	Moderate OSA *N* = 975^2^	Severe OSA *N* = 2955^2^	*p*-value^3^
Age	5,885	42 (34, 52)	37 (30, 47)	41 (33, 52)	44 (36, 55)	43 (35, 53)	<0.001
Sex	5,885						<0.001
Female		1,159 (20%)	394 (37%)	222 (25%)	200 (21%)	343 (12%)	
Male		4,726 (80%)	673 (63%)	666 (75%)	775 (79%)	2,612 (88%)	
Weight (kg)	5,885	77 (68, 85)	68 (60, 76)	73 (65, 80)	75 (68, 84)	80 (74, 90)	<0.001
Height (m)	5,885	1.70 (1.65, 1.75)	1.70 (1.62, 1.75)	1.70 (1.65, 1.75)	1.70 (1.65, 1.75)	1.72 (1.68, 1.75)	<0.001
BMI	5,885	26.2 (24.1, 28.9)	23.8 (21.8, 26.0)	25.2 (23.3, 27.4)	26.0 (24.2, 28.1)	27.7 (25.4, 30.1)	<0.001
NC (cm)	5,876	39.0 (37.0, 41.5)	37.0 (34.0, 39.0)	38.0 (36.0, 40.0)	39.0 (37.0, 41.0)	40.5 (39.0, 42.5)	<0.001
WC (cm)	5,885	95 (89, 102)	87 (80, 93)	92 (86, 97)	94 (89, 100)	99 (93, 105)	<0.001
HC (cm)	5,876	100 (96, 105)	96 (93, 101)	99 (95, 103)	100 (96, 104)	103 (99, 107)	<0.001
Systolic blood pressure	5,451	120 (120, 133)	120 (119, 122)	120 (120, 120)	122 (116, 134)	126 (120, 137)	<0.001
Diastolic blood pressure	5,451	80 (76, 85)	80 (76, 80)	80 (80, 80)	80 (73, 85)	80 (77, 90)	<0.001
Smoking	5,885	1,062 (18%)	195 (18%)	141 (16%)	168 (17%)	558 (19%)	0.2
Alcohol consumption	5,885	2,948 (50%)	502 (47%)	434 (49%)	494 (51%)	1,518 (51%)	0.088
Fasting Blood Glucose (mg/dL)	5,885	94 (88, 103)	91 (84, 96)	92 (86, 99)	94 (88, 103)	97 (90, 108)	<0.001
Fasting insulin (μU/mL)	5,885	11 (7, 16)	8 (5, 11)	9 (6, 13)	10 (7, 15)	13 (9, 18)	<0.001
HOMA-IR	5,885	2.51 (1.61, 3.92)	1.71 (1.13, 2.55)	2.08 (1.37, 3.20)	2.44 (1.64, 3.63)	3.09 (2.05, 4.74)	<0.001
TC (mg/dL)	5,885	181 (160, 206)	169 (148, 192)	178 (158, 203)	183 (162, 207)	187 (164, 211)	<0.001
TG (mg/dL)	5,885	140 (97, 204)	105 (69, 150)	127 (89, 178)	143 (101, 204)	158 (112, 229)	<0.001
HDL-C (mg/dL)	5,885	40 (35, 46)	43 (37, 50)	41 (36, 48)	40 (35, 47)	39 (34, 44)	<0.001
LDL-C (mg/dL)	5,885	114 (94, 134)	102 (83, 121)	113 (93, 131)	115 (96, 135)	118 (97, 138)	<0.001
ApoA1 (mg/dL)	5,885	105 (94, 118)	107 (96, 122)	106 (94, 120)	105 (94, 118)	104 (94, 116)	<0.001
ApoB (mg/dL)	5,885	84 (72, 97)	74 (63, 88)	82 (71, 94)	84 (72, 98)	87 (76, 100)	<0.001
ApoE (mg/dL)	5,885	4.20 (3.44, 5.26)	3.77 (3.13, 4.67)	4.02 (3.38, 4.92)	4.24 (3.46, 5.22)	4.43 (3.59, 5.63)	<0.001
TyG index	5,885	8.81 (8.42, 9.23)	8.45 (7.99, 8.87)	8.68 (8.28, 9.06)	8.84 (8.46, 9.22)	8.97 (8.60, 9.37)	<0.001
TyG-BMI index	5,885	234 (207, 263)	201 (178, 228)	219 (198, 244)	231 (208, 255)	249 (225, 277)	<0.001
METS-IR	5,885	42 (37, 48)	36 (31, 41)	40 (35, 44)	41 (37, 46)	45 (40, 50)	<0.001
Metabolic_Syndrome	5,451	2,198 (40%)	202 (20%)	141 (19%)	405 (44%)	1,450 (52%)	<0.001
ODI	5,885	30 (9, 58)	2 (1, 4)	10 (7, 13)	22 (18, 27)	57 (43, 72)	<0.001
MAI	5,885	23 (13, 41)	13 (8, 20)	17 (11, 26)	20 (13, 30)	35 (20, 53)	<0.001
Min SpO_2_ (%)	5,885	81 (70, 89)	93 (90, 95)	87 (84, 91)	83 (78, 87)	71 (63, 79)	<0.001
CT90 (%)	5,885	3 (0, 15)	0 (0, 0)	0 (0, 1)	2 (0, 4)	14 (5, 31)	<0.001
TST (min)	5,885	407 (354, 447)	393 (332, 436)	397 (342, 435)	400 (348, 442)	417 (368, 454)	<0.001
SE (ratio)	5,885	0.85 (0.74, 0.93)	0.82 (0.70, 0.90)	0.82 (0.71, 0.90)	0.85 (0.73, 0.92)	0.87 (0.77, 0.94)	<0.001
REM/TST (%)	5,885	11 (6, 15)	12 (7, 17)	11 (6, 15)	10 (6, 14)	10 (6, 14)	<0.001
N1/TST (%)	5,885	15 (8, 24)	14 (8, 21)	14 (7, 23)	16 (8, 24)	16 (8, 25)	<0.001
N3/TST (%)	5,885	13 (6, 20)	15 (8, 22)	15 (8, 21)	13 (7, 20)	11 (4, 18)	<0.001

1 *N* Non-missing.

2 Median (Q1, Q3); *n* (%).

3 Kruskal-Wallis rank sum test; Pearson’s Chi-squared test.

### 3.2 Spearman correlation and multiple linear regression analyses

**Figure 1:**
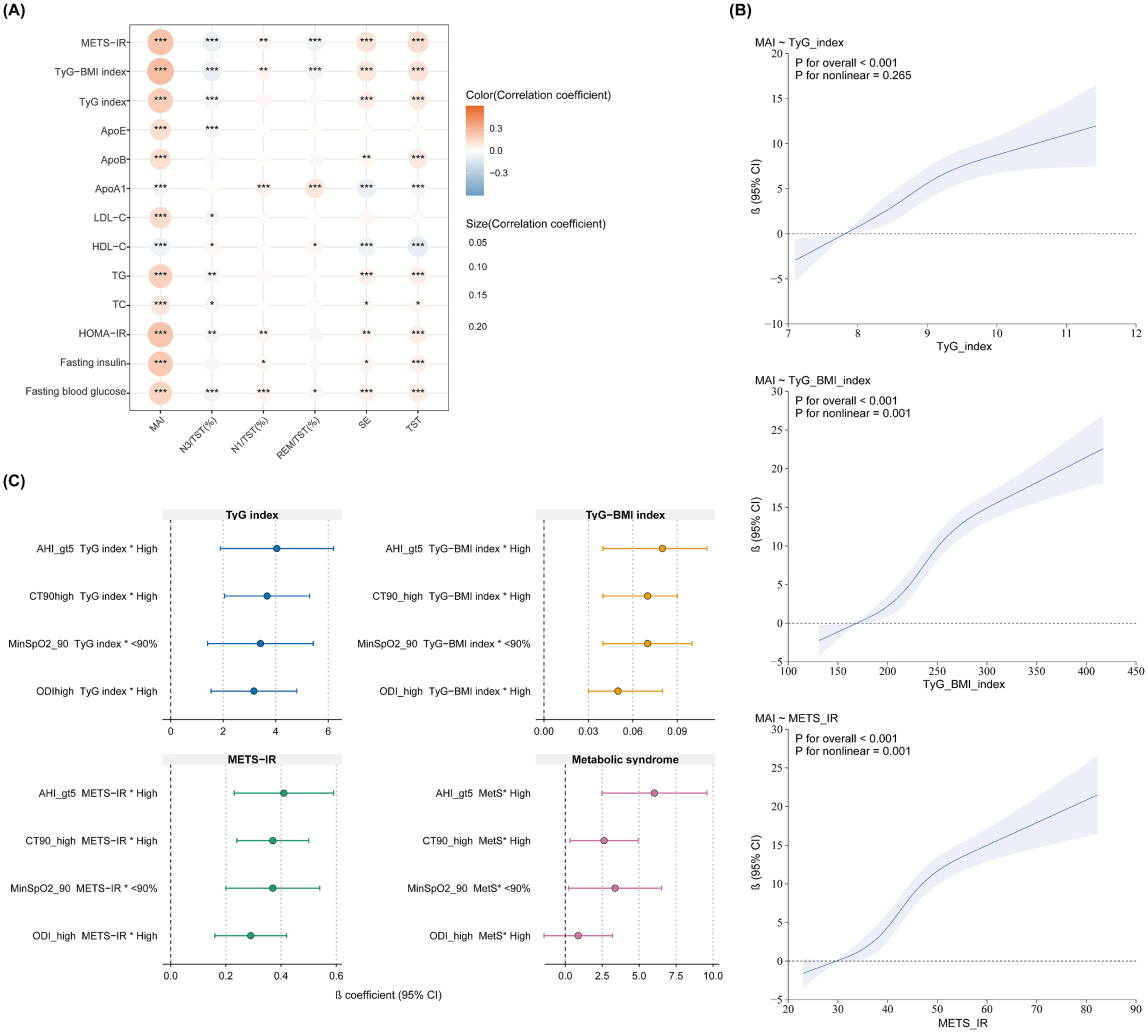
Associations between glucose-lipid metabolism and sleep structure. **(A)** Spearman correlation between glucose–lipid metabolism parameters and sleep structure. The horizontal axis represents sleep structure variables, and the vertical axis represents glucose–lipid metabolism indices. The size and color of the circles indicate the magnitude and direction of the correlation coefficients, respectively. Statistical significance is denoted by asterisks. **(B)** Nonlinear relationships between glucose–lipid composite indices and MAI. Restricted cubic spline (RCS) models were used to evaluate the nonlinear associations of TyG index, TyG-BMI index, and METS-IR with MAI. Models were adjusted for age, sex, body mass index (BMI), smoking status, and alcohol consumption. **(C)** Interaction effects on MAI. Forest plots show the interaction effects between glucose–lipid metabolic indices and obstructive sleep apnea (OSA)-related hypoxia indicators on MAI after dichotomization.

Spearman correlation analysis revealed significant associations between glucose–lipid metabolic indices and multiple sleep architecture parameters (Figure 1A), with the strongest correlation observed for MAI (all *p* < 0.05).

In multiple linear regression analyses adjusted for age, sex, BMI, smoking, and alcohol consumption (Table 2), the TyG index was independently and positively associated with TST (*β* = 6.4, *p* < 0.001), SE (*β* = 0.010, *p* = 0.002), and MAI (*β* = 4.0, p < 0.001). The TyG-BMI index and METS-IR showed similar positive associations with TST, SE, and MAI (all *p* < 0.001), showing patterns similar to those observed for the TyG index.

**Table 2:** Multivariate linear regression analysis of the association between glucose–LipidIndices, metabolic syndrome and sleep parameters.

Characteristic	TyG index			TyG-BMI index			METS-IR			MetS (ref = No MetS)		
	Beta	95% CI	*p*-value	Beta	95% CI	*p*-value	Beta	95% CI	*p*-value	Beta	95% CI	*p*-value
TST	6.4	3.5, 9.3	**<0.001**	0.15	0.11, 0.19	**<0.001**	0.80	0.59, 1.0	**<0.001**	2.2	−1.8, 6.2	0.3
SE	0.010	0.004, 0.016	**0.002**	0.0002	0.0002, 0.0003	**<0.001**	0.001	0.001, 0.002	**<0.001**	0.007	−0.002, 0.015	0.13
REM/TST (%)	0.03	−0.24, 0.30	0.8	−0.0076	−0.0113, −0.0038	**<0.001**	−0.04	−0.06, −0.02	**<0.001**	−0.46	−0.83, −0.09	**0.015**
N1/TST (%)	−0.15	−0.68, 0.37	0.6	0.008	0.001, 0.016	**0.025**	0.04	0.00, 0.08	0.053	−0.01	−0.74, 0.72	>0.9
N3/TST (%)	0.30	−0.17, 0.77	0.2	−0.01	−0.02, −0.01	**<0.001**	−0.07	−0.11, −0.04	**<0.001**	−0.31	−0.96, 0.34	0.4
MAI	4.0	3.1, 4.9	**<0.001**	0.11	0.10, 0.13	**<0.001**	0.53	0.47, 0.60	**<0.001**	4.7	3.5, 6.0	**<0.001**

CI, confidence interval.

All models were adjusted for age, gender, BMI, smoking, and alcohol consumption. Due to the inclusion of BMI in the TyG-BMI index and METS-IR models, BMI was not adjusted for again in these models.

MetS, metabolic syndrome. Bold values indicate statistically significant p-values (*p* < 0.05).

Metabolic syndrome was associated with a reduced proportion of REM sleep and an increased MAI, whereas no significant associations were observed with TST or other sleep stage parameters. These findings further support an association between metabolic dysregulation and sleep fragmentation.

Overall, glucose–lipid metabolic abnormalities and metabolic syndrome were associated with alterations in sleep architecture, particularly increased MAI.

### 3.3 RCS and threshold effect analyses

To investigate the dose–response relationships between glucose–lipid metabolic indices and MAI, RCS analyses were performed. As shown in Figure 1B, after adjustment for key covariates, the TyG index exhibited a linear association with MAI (p_overall < 0.001; p_non-linearity = 0.265), whereas both the TyG-BMI index and METS-IR demonstrated significant nonlinear associations with MAI (both *p* < 0.01).

Further threshold effect analyses confirmed the presence of nonlinear relationships (Supplementary Tables S1). For the TyG-BMI index, when values were below 310.31, each one-unit increase was associated with a 0.13 increase in MAI (*p* < 0.001); however, this association became nonsignificant above the threshold (*β* = 0.016, *p* = 0.574). Similarly, for METS-IR, values below 57.25 were associated with a 0.60 increase in MAI per unit increase (*p* < 0.001), whereas no significant association was observed beyond the threshold (*β* = 0.147, *p* = 0.322).

*F*-tests indicated that the segmented regression model provided a significantly better fit than the linear model, supporting the existence of threshold effects (all *p* < 0.01).

### 3.4 Interaction effects

To investigate whether OSA severity and hypoxia modify the associations between glucose–lipid metabolic dysregulation, metabolic syndrome, and MAI, both multiplicative and additive interaction analyses were conducted.

In multivariable linear regression models incorporating interaction terms between continuous variables (Table 3), significant positive multiplicative interactions were observed between AHI and the TyG index, METS-IR, and metabolic syndrome (all *p* < 0.05), indicating that the association between metabolic indices and MAI became stronger with increasing respiratory event frequency. ODI also showed significant interactions with all three composite metabolic indices and metabolic syndrome (all *p* < 0.01). In contrast, no significant interactions were observed between Min SpO₂ and any metabolic indicators. For the hypoxic burden indicator CT90, significant interactions with negative coefficients were detected for the TyG-BMI index, METS-IR, and metabolic syndrome (all *p* < 0.01), whereas no significant interaction was found between CT90 and the TyG index.

**Table 3:** Multivariate linear regression analysis of multiplicative interaction effects between glucose–lipidindices, metabolic syndrome, and MAI.

Interaction terms		MAI		
		Beta	95% CI	*p*-value
AHI	TyG index * AHI	0.05	0.02, 0.08	**<0.001**
	TyG-BMI index * AHI	0.0004	−0.00002, 0.00077	0.061
	METS-IR * AHI	0.0026	0.0006, 0.0047	**0.013**
	MetS * AHI	0.05	0.01, 0.09	**0.010**
ODI	TyG index * ODI	0.06	0.03, 0.08	**<0.001**
	TyG-BMI index * ODI	0.0005	0.0002, 0.0009	**0.005**
	METS-IR * ODI	0.003	0.001, 0.005	**<0.001**
	MetS * ODI	0.07	0.03, 0.11	**<0.001**
Min_SpO_2_	TyG index * Min_SpO_2_	−0.04	−0.10, 0.02	0.2
	TyG-BMI index * Min_SpO_2_	−0.00029	−0.00112, 0.00054	0.5
	METS-IR * Min_SpO_2_	−0.0029	−0.0072, 0.0014	0.2
	MetS * Min_SpO_2_	0.02	−0.07, 0.11	0.7
CT90	TyG index * CT90	−0.02	−0.06, 0.03	0.5
	TyG-BMI index * CT90	−0.00131	−0.00198, −0.00065	<0.001
	METS-IR * CT90	−0.0061	−0.0096, −0.0026	<0.001
	MetS * CT90	−0.10	−0.17, −0.04	0.002

The interaction terms represent the multiplicative interactions between AHI, ODI, Min SpO₂, CT90, and Glucose–Lipidindices (TyG index, TyG-BMI index, METS-IR) as well as MetS.

MetS, metabolic syndrome. Bold values indicate statistically significant p-values (*p* < 0.05).

After dichotomizing OSA severity and hypoxic variables, multiplicative interaction analyses yielded broadly consistent results (Figure 1C). The association between TyG and MAI was stronger among participants with AHI > 5 (*β* = 4.04, 95% CI 1.89–6.20, *p* < 0.001). The corresponding interaction effects for the TyG-BMI index and METS-IR were 0.08 (95% CI 0.04–0.11) and 0.41 (95% CI 0.23–0.59), respectively (both *p* < 0.001). Similar patterns were observed for ODI, CT90, and Min SpO₂. Metabolic syndrome also exhibited significant interactions with AHI > 5 (*β* = 6.03, 95% CI 2.48–9.58), elevated CT90 (*β* = 2.63, 95% CI 0.31–4.94), and Min SpO₂ < 90% (*β* = 3.37, 95% CI 0.22–6.52), but not with ODI.

Further additive interaction analyses (logistic regression) demonstrated significant positive additive interactions between high TyG, TyG-BMI, or METS-IR and elevated AHI or nocturnal hypoxia, with RERI values >0 and *S* values >1. The combined exposure also yielded multiplicative ORs of approximately 4.07–5.91 (Supplementary Tables S2). Similar trends were observed for metabolic syndrome. Overall, the associations between glucose–lipid metabolic indices and MAI were stronger among participants with greater OSA severity and nocturnal hypoxia.

### 3.5 Sensitivity analyses

Stratified analyses by sex and age demonstrated consistent positive associations of glucose–lipid metabolic indices and MetS with MAI (Supplementary Tables S3).

In addition, results remained robust after controlling for multiple testing using the BH FDR correction. The associations between glucose–lipid metabolic indices, MetS, and MAI were largely consistent with the primary analyses after FDR adjustment, with key associations remaining statistically significant (*q* < 0.05), supporting the stability of the main findings (Supplementary Tables S4–S7).

### 3.6 Exploration of CIH-induced glucose–lipid metabolic reprogramming in brainstem cells

Given that the association between glucose–lipid metabolic abnormalities and sleep fragmentation was stronger among individuals with more severe nocturnal hypoxia, we further explored whether CIH was associated with alterations in metabolic pathway activity in brainstem cells.

**Figure 2:**
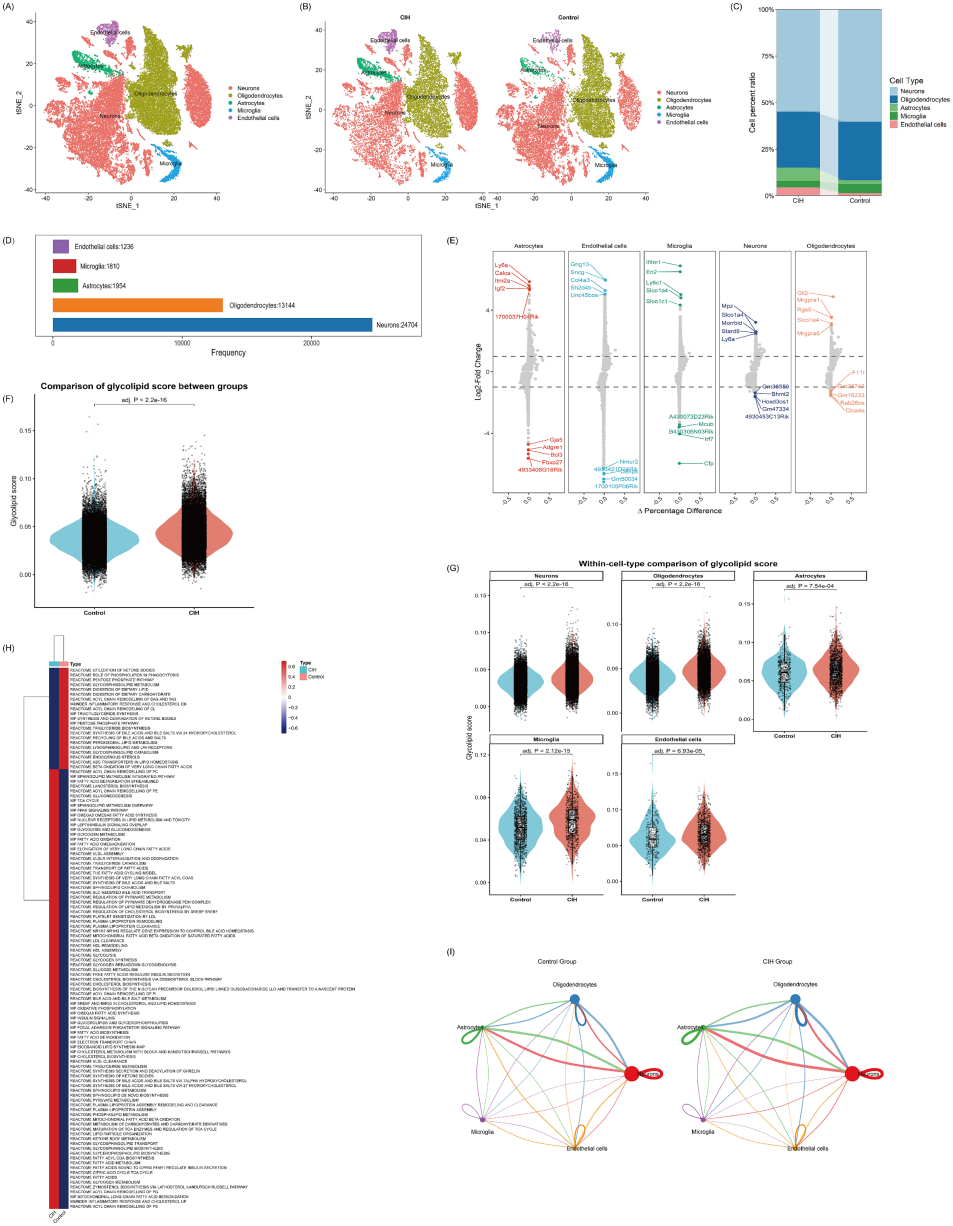
CIH induces glucose–lipid metabolic reprogramming and enhances intercellular communication in the mouse brainstem. **(A)** t-distributed stochastic neighbor embedding (t-SNE) plot showing the overall cellular landscape of the mouse brainstem based on single-nucleus RNA sequencing data. **(B)** t-SNE plot colored by groups (Control vs. CIH), illustrating the distribution of cells under different conditions. **(C)** Bar plot showing the relative proportions of major brainstem cell types in Control and CIH groups. **(D)** Bar plot showing the absolute cell counts of each identified cell type. **(E)** Volcano plot of differentially expressed genes (DEGs) between CIH and Control groups. **(F)** Comparison of glucose–lipid metabolism scores between groups. Glucose–lipid metabolic activity scores were calculated using the AddModuleScore method based on curated gene sets. **(G)** Within-cell-type comparison of glucose-lipid metabolism scores. **(H)** Heatmap of glucose–lipid metabolism pathway activity based on gene set variation analysis (GSVA). **(I)** Cell–cell communication networks inferred by CellChat analysis.

Cell types were annotated using SingleR, identifying five major neural cell populations in the brainstem, including neurons, oligodendrocytes, astrocytes, microglia, and endothelial cells (Figures 2A,B). Differential expression analysis revealed that glucose–lipid metabolism-related genes, such as *Igf2* (astrocytes) and *Stard8* (neurons), were significantly upregulated in the CIH group (Figure 2E).

Subsequently, using a curated glucose–lipid metabolism gene set, we assessed metabolic activity at the single-nucleus level. Cells from the CIH group showed higher glucose–lipid metabolic module scores than controls (adj. *p* < 2.2 × 10^−16^) (Figure 2F). Cell type–specific analyses further demonstrated statistically significant metabolic activation in multiple brainstem cell populations, including neurons (adj. *p* < 2.2 × 10^−16^), oligodendrocytes (adj. *p* < 2.2 × 10^−16^), astrocytes (adj. *p* = 7.54 × 10^−4^), microglia (adj. *p* = 2.12 × 10^−14^), and endothelial cells (adj. *p* = 6.93 × 10^−5^) (Figure 2G). Notably, neurons and oligodendrocytes exhibited the most pronounced metabolic alterations.

GSVA further confirmed that most glucose–lipid metabolic pathways were activated in the CIH group, whereas these pathways remained relatively suppressed in the control group, suggesting widespread alterations in metabolic pathway activity in the brainstem under CIH (Figure 2H).

In addition, cell–cell communication network analysis demonstrated a marked increase in intercellular signaling in the CIH group (Figure 2I). The most prominent change was enhanced communication between astrocytes and neurons, suggesting increased astrocyte–neuron signaling under CIH conditions.

While these findings are exploratory and do not directly test the causal link between metabolic dysregulation and sleep fragmentation, they suggest that chronic hypoxia was associated with altered metabolic pathway activity and increased intercellular communication in brainstem cells.

### 3.7 Exploratory identification of candidate genes

**Figure 3:**
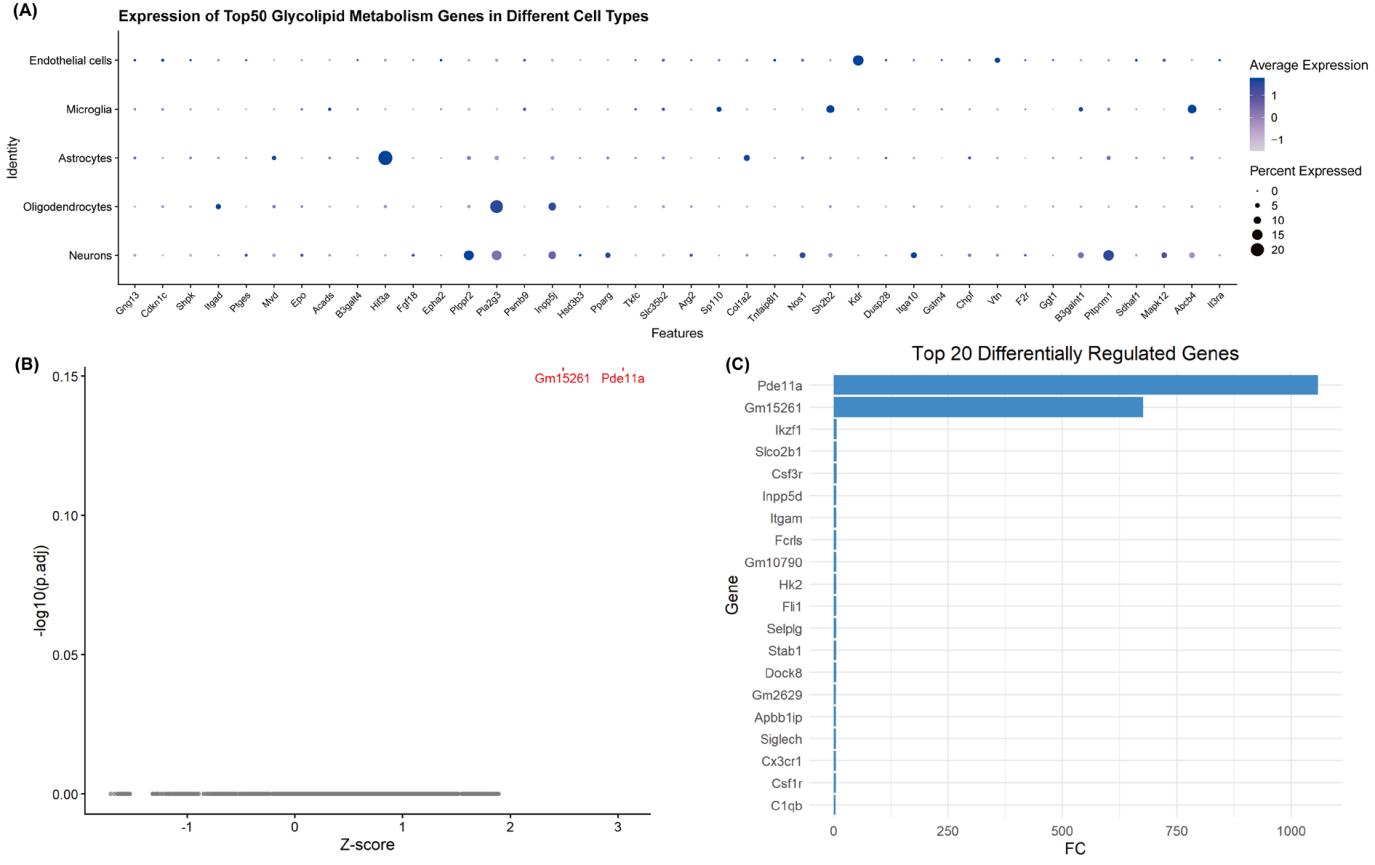
Identification of glucose-lipid metabolism–related downstream targets and virtual gene perturbation analysis. **(A)** Bubble plot showing the expression patterns of the top 50 glucose-lipid metabolism–related differentially expressed genes across major brainstem cell types. **(B)** Bar plot illustrating the top 20 differentially regulated genes identified through virtual gene knockout analysis using the scTenifoldKnk algorithm. **(C)** Volcano plot of gene regulatory changes following virtual knockout of *Inpp5j*. Each point represents a gene, with the *x*-axis indicating regulatory *Z*-score and the y-axis representing –log10 adjusted *p* values. Significantly altered genes are highlighted in red.

By intersecting the cell type–specific differential expression results with the previously defined glucose–lipid metabolism gene set, the top 50 differentially expressed glucose–lipid–related genes were identified (Figure 3A). Among these, *Pla2g3* and *Inpp5j* were selected, together with *Igf2* and *Stard8* identified in the previous differential expression analysis, yielding four candidate glucose–lipid–related genes for subsequent in virtual gene knockout analysis.

Notably, simulated knockout of *Inpp5j* predicted changes in *Pde11a*-related regulatory patterns, a gene closely associated with sleep and micro-arousal states (Figures 3B,C).

These exploratory findings nominate *Inpp5j* and *Pde11a* as candidate genes for future investigation of hypoxia-related brainstem responses. However, the results should be considered hypothesis-generating, do not establish a molecular pathway, and require further experimental validation.

## 4 Discussion

This study, based on a large clinical cohort and integrated single-nucleus transcriptomic analysis, systematically investigated the relationship between glucose–lipid metabolic dysregulation and sleep fragmentation in OSA. We found that multiple glucose–lipid composite metabolic indices increased with OSA severity and were positively associated with MAI. These associations were further confirmed by multivariable analyses, in which TyG index, TyG-BMI, and METS-IR were independently linked to MAI. Dose–response analyses demonstrated a linear association between TyG index and MAI, while TyG-BMI and METS-IR showed nonlinear relationships with threshold effects. Importantly, the associations between glucose–lipid metabolic indices and MAI were stronger among participants with greater OSA severity and nocturnal hypoxia, consistent with effect modification. Exploratory single-nucleus analysis of a CIH brainstem model suggested alterations in glucose–lipid metabolic activity and intercellular communication in brainstem cells. In virtual analyses highlighted *Inpp5j* as a candidate gene for future investigation. These findings provide exploratory evidence of hypoxia-associated metabolic changes in the brainstem, without establishing causal effects on sleep fragmentation.

TyG index, TyG-BMI index, and METS-IR are non–insulin-based surrogate markers of insulin resistance that integrate information on glucose, lipid profiles, and body composition. Compared with HOMA-IR, they are more cost-effective, easily accessible, and demonstrate favorable predictive performance, making them particularly suitable for evaluating glucose–lipid metabolic status in large-scale epidemiological studies (10, 11, 35, 36). In the present study, we further observed that these composite metabolic indices showed consistent patterns with metabolic syndrome across the main analyses, all of which were significantly associated with sleep fragmentation, suggesting good concordance and robustness in reflecting metabolic dysregulation. Recent studies have reported that TyG-related indices are positively associated with OSA risk, disease severity, and nocturnal hypoxia burden, supporting their utility as accessible markers of metabolic dysfunction in OSA populations (15, 16, 37, 38). These observations, together with our findings, support a close relationship between glucose–lipid metabolic abnormalities and OSA and further highlight the utility of TyG-related indices in characterizing metabolic disturbances associated with SDB. Compared with the categorical definition of metabolic syndrome, these continuous-variable-based indices may more sensitively capture early metabolic alterations and exhibit greater stability and practicality in large cohort studies, thereby enhancing the reliability and interpretability of the findings (35, 39). As an important comorbidity of OSA, glucose–lipid metabolic dysregulation not only contributes to the development of insulin resistance and cardiovascular risk but may also exert adverse effects on the nervous system through disturbances in energy metabolism and inflammatory processes (3, 24, 40). Previous studies have suggested that metabolic abnormalities may induce oxidative stress, mitochondrial dysfunction, and neuroinflammation, thereby exerting potential neurotoxic effects on the central nervous system and disrupting the stability of sleep-related neural circuits (41–43). In this context, our findings further suggest that glucose–lipid metabolic dysregulation is associated with sleep architecture disruption, beyond being a comorbid feature of OSA.

Importantly, the relationship between OSA and glucose–lipid metabolic dysregulation is likely bidirectional. Accumulating evidence suggests that OSA-related intermittent hypoxia, sleep fragmentation, sympathetic overactivation, and systemic inflammation may in turn promote insulin resistance, dyslipidemia, and broader metabolic dysfunction (44–46). Therefore, the observed associations in this study may reflect a reciprocal relationship between metabolic dysregulation and sleep disturbance rather than a unidirectional effect. Given the cross-sectional design of this study, the temporal sequence and causal direction between these processes cannot be determined.

Notably, MAI is not merely a PSG parameter but may reflect broader alterations in sleep continuity and sleep regulatory processes. Sleep fragmentation may be linked to metabolic dysregulation through multiple mechanisms. On the one hand, peripheral metabolic abnormalities may indirectly disrupt sleep homeostasis by inducing inflammatory responses and endocrine alterations. On the other hand, metabolic dysregulation may directly affect neuronal excitability and neurotransmitter release by altering cerebral energy metabolism, thereby impairing sleep–wake regulatory networks (47–50). The dose–response relationships observed in this study further support a continuous association between glucose–lipid metabolic dysregulation and sleep fragmentation. In contrast, the nonlinear and threshold effects observed for the TyG-BMI index and METS-IR suggest that, beyond a certain degree of glucose–lipid metabolic impairment, the impact on sleep architecture may plateau or be modulated by additional physiological regulatory mechanisms (16, 17).

Considering that the core pathophysiological feature of OSA lies in repetitive cycles of hypoxia and reoxygenation, analyses focusing solely on glucose–lipid metabolic factors are insufficient to fully elucidate its complex mechanisms. Therefore, this study systematically evaluated their synergistic effects from both multiplicative and additive interaction perspectives. The results demonstrated that OSA severity and hypoxic burden (ODI, CT90, and Min SpO₂) significantly strengthened the association between glucose–lipid metabolic dysregulation and MAI, suggesting that hypoxia may as an important effect modifier in this relationship. It is important to emphasize that this study primarily focused on the indirect role of hypoxia in sleep fragmentation through potentiating the effects of metabolic dysregulation, rather than solely examining its direct effects. However, this does not preclude the well-established direct impact of hypoxia on sleep architecture, which remains a dominant mechanism in OSA pathophysiology (51–53). Collectively, these findings suggest that glucose–lipid metabolic dysregulation and hypoxia may jointly contribute to sleep architecture disruption through interactive effects, highlighting a key component of OSA pathophysiology.

In the interaction analysis using continuous hypoxemia measures, the CT90 × metabolic index interaction term showed a negative coefficient, likely reflecting the marginal and potentially non-linear nature of CT90 across its wide physiological range (Table 3; Supplementary Table S6). To enhance interpretability, we additionally performed analyses using dichotomized hypoxemia indicators, in which CT90, ODI, and minimum SpO₂ consistently showed positive interaction effects, indicating stronger associations between metabolic dysregulation and MAI under more severe hypoxemia (Figure 1C; Supplementary Table S7). Overall, these results suggest that clinically stratified hypoxemia measures may better capture the effect modification of nocturnal hypoxia on the relationship between metabolic status and sleep fragmentation.

Previous studies have suggested that neurons are highly dependent on energy metabolism and that their excitability and synaptic transmission require tightly regulated metabolic support. Glucose–lipid metabolic dysregulation can impair ATP production, mitochondrial function, and redox homeostasis, thereby altering neuronal activity and disrupting sleep–wake regulatory networks (54–56). In line with this, our exploratory single-nucleus analyses showed increased glucose–lipid metabolic activity across multiple brainstem cell types under CIH conditions, suggesting widespread alterations in metabolic pathway activity. Such changes may help maintain cellular function under hypoxic stress but could also increase metabolic burden and oxidative stress (57). Recent proteomic studies have similarly reported systemic metabolic remodeling in OSA, including alterations in erythrocyte glycolytic pathways and the Rapoport–Luebering shunt. Although derived from peripheral blood rather than brain tissue, these findings support the presence of hypoxia-associated metabolic remodeling observed in our analyses (58). Notably, *Inpp5j* was identified as a candidate lipid metabolism–related gene and may potentially regulate sleep-related molecules such as *Pde11a*, suggesting a possible link between metabolic alterations and neural circuit regulation (59, 60). Collectively, these exploratory findings suggest that CIH is associated with alterations in glucose–lipid metabolic pathway activity in brainstem cells, providing hypotheses for future studies investigating the links among hypoxia, metabolic dysregulation, and sleep fragmentation.

Although this study benefits from a relatively large sample size and multi-level analytical approaches, several limitations should be acknowledged. First, the cross-sectional design precludes any inference of causality between glucose–lipid metabolic dysregulation and sleep fragmentation. Although multivariable adjustment and dose–response analyses were applied to enhance the robustness of the findings, prospective cohort studies and interventional trials are still required to further clarify their causal relationship. Second, the study population consisted of individuals referred for snoring and suspected OSA, which may introduce selection bias and limit the generalizability of the findings to the general population. Third, despite adjustment for multiple confounders, the possibility of residual confounding cannot be excluded, as factors such as physical activity, dietary patterns, and medication use were not fully accounted for, which may have influenced both metabolic status and sleep characteristics. Finally, the exploratory single-nucleus analysis was based on a CIH mouse brainstem model, which reflects hypoxia-related brainstem responses rather than glucose–lipid metabolic dysregulation itself. Accordingly, these findings should be considered exploratory and do not directly support the proposed mechanism linking metabolic dysregulation to sleep fragmentation. Further *in vivo* and *in vitro* studies are needed to validate the key molecular pathways and clarify the relationships among hypoxia, metabolic alterations, and sleep fragmentation.

## 5 Conclusion

In summary, this study, integrating a large clinical cohort with exploratory single-nucleus transcriptomic analysis, demonstrates robust associations between glucose–lipid metabolic dysregulation and OSA-related sleep fragmentation. TyG index, TyG-BMI index, and METS-IR were independently associated with MAI and exhibited clear dose–response and threshold patterns. The associations were stronger among participants with greater OSA severity and nocturnal hypoxia, as reflected in both multiplicative and additive interaction analyses, consistent with hypoxia acting as an effect modifier. Exploratory single-nucleus analyses further indicated that CIH was associated with alterations in glucose–lipid metabolic activity and intercellular communication in brainstem cells. Overall, these findings provide complementary clinical and exploratory molecular evidence linking metabolic dysregulation, hypoxia, and sleep fragmentation in OSA.

## Data Availability

Publicly available datasets from the Gene Expression Omnibus (GEO) were analyzed in this study: https://www.ncbi.nlm.nih.gov/geo/, accession GSE256102. Further inquiries can be directed to the corresponding author.
