# Supplementary material for: Glucose–lipid metabolic dysregulation and sleep fragmentation in obstructive sleep apnea: insights from a large-scale cross-sectional study and exploratory hypoxia-related single-nucleus transcriptomic analysis
Source: Front Nutr. 2026 Jul 10;13:1859107. doi: 10.3389/fnut.2026.1859107 (PMC13395911; doi:10.3389/fnut.2026.1859107)
Supplement: Supplementary file 1 [file Data_Sheet_1.ZIP › Supplementary files/Table S1-S7.docx]

| **Threshold Effect Analysis** | **MAI**  **β (95% CI) p-value** |
| --- | --- |
| ​​TyG-BMI Index​​ |  |
| Inflection point (K) | ​​310.31​​ |
| < K slope | ​​0.129 (0.114, 0.145) **<0.001**​​ |
| > K slope | ​​0.016 (-0.040, 0.072) 0.574​​ |
| F-test (ANOVA) | ​​**<0.001**​ |
| ​​****METS-IR****​​ |  |
| Inflection point (K) | ​​57.25​​ |
| < K slope | ​​0.597 (0.515, 0.679) **<0.001​**​ |
| > K slope | ​​0.147 (-0.144, 0.438) 0.322​​ |
| F-test (ANOVA) | ​​**0.006**​​ |

**Table S1. Threshold Effect Analysis of TyG-BMI Index and **METS-IR** on MAI**

**Table S2. Additive and Multiplicative Interaction Effects of Glucose–Lipid Indices, MetS on MAI**

| **Exposure / Modifier** | **RERI (est [95% CI])** | **APAB (est [95% CI])** | **S (est [95% CI])** | **Multiplicative (est [95% CI])** |
| --- | --- | --- | --- | --- |
| TyG high + AHI_gt5 | 1.494 (0.885, 2.104) | 0.289 (0.184, 0.394) | 1.559 (1.268, 1.915) | 5.170 (4.185, 6.418) |
| TyG high + ODI_high | 0.539 (-0.368, 1.446) | 0.091 (-0.056, 0.239) | 1.123 (0.921, 1.369) | 5.915 (5.036, 6.959) |
| TyG high + CT90_high | 1.095 (0.286, 1.904) | 0.194 (0.065, 0.324) | 1.309 (1.069, 1.603) | 5.638 (4.796, 6.638) |
| TyG high + MinSpO2_90 | 0.937 (0.388, 1.486) | 0.209 (0.093, 0.324) | 1.368 (1.115, 1.678) | 4.486 (3.697, 5.462) |
| TyG-BMI high + AHI_gt5 | 1.651 (0.811, 2.492) | 0.287 (0.169, 0.404) | 1.531 (1.236, 1.897) | 5.761 (4.570, 7.291) |
| TyG-BMI high + ODI_high | 0.790 (-0.228, 1.807) | 0.136 (-0.025, 0.297) | 1.196 (0.952, 1.504) | 5.809 (4.820, 7.012) |
| TyG-BMI high + CT90_high | 1.264 (0.359, 2.170) | 0.232 (0.091, 0.373) | 1.397 (1.106, 1.764) | 5.449 (4.522, 6.576) |
| TyG-BMI high + MinSpO2_90 | 1.259 (0.602, 1.915) | 0.276 (0.156, 0.396) | 1.548 (1.226, 1.955) | 4.556 (3.690, 5.638) |
| METS-IR high + AHI_gt5 | 1.153 (0.391, 1.914) | 0.218 (0.092, 0.343) | 1.366 (1.109, 1.684) | 5.298 (4.201, 6.706) |
| METS-IR high + ODI_high | 0.251 (-0.699, 1.200) | 0.048 (-0.130, 0.226) | 1.063 (0.843, 1.342) | 5.210 (4.338, 6.267) |
| METS-IR high + CT90_high | 0.768 (-0.061, 1.597) | 0.158 (0.003, 0.313) | 1.248 (0.983, 1.584) | 4.867 (4.052, 5.853) |
| METS-IR high + MinSpO2_90 | 0.922 (0.337, 1.506) | 0.226 (0.100, 0.353) | 1.429 (1.128, 1.809) | 4.072 (3.304, 5.029) |
| MetS + AHI_gt5 | 1.692 (0.944, 2.440) | 0.296 (0.185, 0.406) | 1.558 (1.265, 1.919) | 5.723 (4.600, 7.156) |
| MetS + ODI_high | 0.369 (-0.541, 1.280) | 0.065 (-0.090, 0.220) | 1.086 (0.886, 1.331) | 5.674 (4.802, 6.717) |
| MetS + CT90_high | 0.699 (-0.135, 1.533) | 0.130 (-0.014, 0.274) | 1.190 (0.967, 1.465) | 5.374 (4.548, 6.361) |
| MetS + MinSpO2_90 | 0.889 (0.248, 1.529) | 0.192 (0.063, 0.321) | 1.324 (1.066, 1.644) | 4.630 (3.797, 5.663) |

**Table S3. Sensitivity Analysis of the Association between Glucose–Lipid Indices, MetS, and MAI Stratified by Sex and Age**

|  | **TyG** | | | **TyG-BMI** | | | **METS-IR** | | | **MetS (ref = No MetS)** | | |
| --- | --- | --- | --- | --- | --- | --- | --- | --- | --- | --- | --- | --- |
| **Characteristic** | **Beta** | **95% CI** | **p-value** | **Beta** | **95% CI** | **p-value** | **Beta** | **95% CI** | **p-value** | **Beta** | **95% CI** | **p-value** |
| **Sex** |  |  |  |  |  |  |  |  |  |  |  |  |
| Male | 4.2 | 3.2, 5.2 | **<0.001** | 0.13 | 0.11, 0.14 | **<0.001** | 0.57 | 0.50, 0.65 | **<0.001** | 4.9 | 3.5, 6.3 | **<0.001** |
| Female | 3.0 | 1.2, 4.7 | **0.001** | 0.07 | 0.05, 0.09 | **<0.001** | 0.39 | 0.27, 0.50 | **<0.001** | 3.7 | 0.90, 6.4 | **0.009** |
| **Age** |  |  |  |  |  |  |  |  |  |  |  |  |
| 18-33 | 3.3 | 1.5, 5.1 | **<0.001** | 0.11 | 0.09, 0.13 | **<0.001** | 0.53 | 0.41, 0.65 | **<0.001** | 3.8 | 1.1, 6.4 | **0.005** |
| 34-41 | 2.6 | 0.83, 4.3 | **0.004** | 0.12 | 0.10, 0.15 | **<0.001** | 0.52 | 0.40, 0.65 | **<0.001** | 5.3 | 2.9, 7.8 | **<0.001** |
| 42-51 | 5.4 | 3.6, 7.3 | **<0.001** | 0.13 | 0.10, 0.16 | **<0.001** | 0.64 | 0.48, 0.79 | **<0.001** | 6.6 | 4.0, 9.2 | **<0.001** |
| 52-87 | 4.7 | 2.9, 6.4 | **<0.001** | 0.09 | 0.06, 0.11 | **<0.001** | 0.41 | 0.28, 0.55 | **<0.001** | 3.2 | 0.92, 5.4 | **0.006** |
| Abbreviation: CI = Confidence Interval | | | | | | | | | |  | | |

**Table S4. Multivariate Linear Regression Analysis of the Association between Glucose–Lipid Indices, MetS and MAI with FDR-Adjusted q Values**

| **Characteristic** | **Outcome** | **p value** | **q value** |
| --- | --- | --- | --- |
| TyG | MAI | **<0.001** | **<0.001** |
| TyG-BMI | MAI | **<0.001** | **<0.001** |
| METS-IR | MAI | **<0.001** | **<0.001** |
| MetS | MAI | **<0.001** | **<0.001** |

**Table S5. Restricted Cubic Spline Analysis of Glucose–Lipid Indices and MAI with FDR-Adjusted q Values for Overall and Nonlinearity Associations**

| **Characteristic** | **Outcome** | **p overall** | **p nonlinearity** | **q overall** | **q nonlinearity** |
| --- | --- | --- | --- | --- | --- |
| TyG | MAI | **<0.001** | 0.265 | <0.001 | 0.265 |
| TyG-BMI | MAI | **<0.001** | **0.001** | <0.001 | **0.0015** |
| METS-IR | MAI | **<0.001** | **0.001** | <0.001 | **0.0015** |

**Table S6. Multivariate Linear Regression Analysis of Multiplicative Interaction Effects between Glucose–Lipid Indices, MetS, and Continuous Hypoxemia Indicators on MAI with FDR-Adjusted q Values**

| **Characteristic** | **Outcome** | **Modifier** | **Interaction term** | **p value** | **q value** |
| --- | --- | --- | --- | --- | --- |
| TyG | MAI | AHI | TyG index × AHI | **<0.001** | **0.002** |
| TyG-BMI | MAI | AHI | TyG-BMI index × AHI | 0.061 | 0.089 |
| METS-IR | MAI | AHI | METS-IR × AHI | **0.013** | **0.02** |
| MetS | MAI | AHI | MetS × AHI | **0.01** | **0.019** |
| TyG | MAI | ODI | TyG index × ODI | **<0.001** | **<0.001** |
| TyG-BMI | MAI | ODI | TyG-BMI index × ODI | **0.005** | **0.009** |
| METS-IR | MAI | ODI | METS-IR × ODI | **<0.001** | **0.002** |
| MetS | MAI | ODI | MetS × ODI | **<0.001** | **0.002** |
| TyG | MAI | Min_SpO2 | TyG index × Min_SpO2 | 0.203 | 0.25 |
| TyG-BMI | MAI | Min_SpO2 | TyG-BMI index × Min_SpO2 | 0.497 | 0.568 |
| METS-IR | MAI | Min_SpO2 | METS-IR × Min_SpO2 | 0.191 | 0.25 |
| MetS | MAI | Min_SpO2 | MetS × Min_SpO2 | 0.653 | 0.653 |
| TyG | MAI | CT90 | TyG index × CT90 | 0.54 | 0.576 |
| TyG-BMI | MAI | CT90 | TyG-BMI index × CT90 | **<0.001** | **<0.001** |
| METS-IR | MAI | CT90 | METS-IR × CT90 | **<0.001** | **0.002** |
| MetS | MAI | CT90 | MetS × CT90 | **0.002** | **0.005** |

**Table S7. Multivariate Linear Regression Analysis of Multiplicative Interaction Effects between Glucose–Lipid Indices, MetS, and Dichotomized Hypoxemia Indicators on MAI with FDR-Adjusted q Values**

| **Characteristic** | **Outcome** | **Modifier** | **Interaction term** | **p value** | **q value** |
| --- | --- | --- | --- | --- | --- |
| TyG | MAI | AHI_gt5 | TyG index × AHI_gt5 | **<0.001** | **<0.001** |
| TyG-BMI | MAI | AHI_gt5 | TyG-BMI index × AHI_gt5 | **<0.001** | **<0.001** |
| METS-IR | MAI | AHI_gt5 | METS-IR × AHI_gt5 | **<0.001** | **<0.001** |
| MetS | MAI | AHI_gt5 | MetS × AHI_gt5 | **<0.001** | **0.001** |
| TyG | MAI | ODI_high | TyG index × ODI_high | **<0.001** | **<0.001** |
| TyG-BMI | MAI | ODI_high | TyG-BMI index × ODI_high | **<0.001** | **<0.001** |
| METS-IR | MAI | ODI_high | METS-IR × ODI_high | **<0.001** | **<0.001** |
| MetS | MAI | ODI_high | MetS × ODI_high | 0.456 | 0.456 |
| TyG | MAI | CT90_high | TyG index × CT90_high | **<0.001** | **<0.001** |
| TyG-BMI | MAI | CT90_high | TyG-BMI index × CT90_high | **<0.001** | **<0.001** |
| METS-IR | MAI | CT90_high | METS-IR × CT90_high | **<0.001** | **<0.001** |
| MetS | MAI | CT90_high | MetS × CT90_high | **0.026** | **0.03** |
| TyG | MAI | MinSpO2_90 | TyG index × MinSpO2_90 | **<0.001** | **0.001** |
| TyG-BMI | MAI | MinSpO2_90 | TyG-BMI index × MinSpO2_90 | **<0.001** | **<0.001** |
| METS-IR | MAI | MinSpO2_90 | METS-IR × MinSpO2_90 | **<0.001** | **<0.001** |
| MetS | MAI | MinSpO2_90 | MetS × MinSpO2_90 | **0.036** | **0.039** |
