# Supplementary figures and images for: Glucose–lipid metabolic dysregulation and sleep fragmentation in obstructive sleep apnea: insights from a large-scale cross-sectional study and exploratory hypoxia-related single-nucleus transcriptomic analysis
Source: Front Nutr. 2026 Jul 10;13:1859107. doi: 10.3389/fnut.2026.1859107 (PMC13395911; doi:10.3389/fnut.2026.1859107)

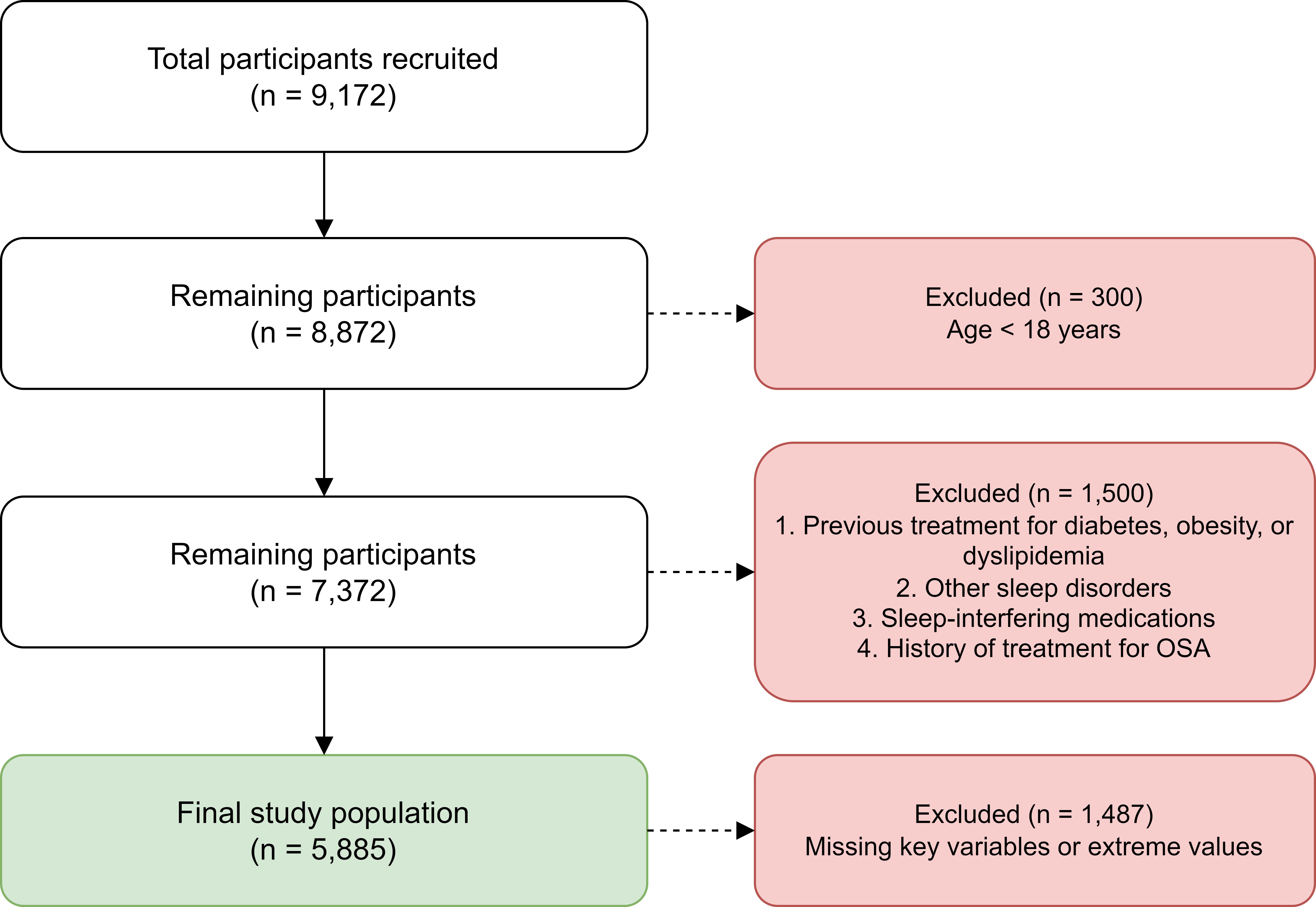

Supplement: Supplementary file 1 [file Data_Sheet_1.ZIP › Supplementary files/Figure S1.jpg]
